# Visualized analysis of developing trends and hot topics in natural disaster research

**DOI:** 10.1371/journal.pone.0191250

**Published:** 2018-01-19

**Authors:** Shi Shen, Changxiu Cheng, Jing Yang, Shanli Yang

**Affiliations:** 1 Key Laboratory of Environmental Change and Natural Disaster, Beijing Normal University, Beijing, China; 2 State Key Laboratory of Earth Surface Processes and Resource Ecology, Beijing Normal University, Beijing, China; 3 Center for Geodata and Analysis, Faculty of Geographical Science, Beijing Normal University, Beijing, China; Oregon State University, UNITED STATES

## Abstract

This study visualized and analyzed the developing trends and hot topics in natural disaster research. 19694 natural disaster-related articles (January 1900 to June 2015) are indexed in the Web of Science database. The first step in this study is using complex networks to visualize and analyze these articles. CiteSpace and Gephi were employed to generate a countries collaboration network and a disciplines collaboration network, and then attached hot topics to countries and disciplines, respectively. The results show that USA, China, and Italy are the three major contributors to natural disaster research. “Prediction model”, “social vulnerability”, and “landslide inventory map” are three hot topics in recent years. They have attracted attention not only from large countries like China but also from small countries like Panama and Turkey. Comparing two hybrid networks provides details of natural disaster research. Scientists from USA and China use image data to research earthquakes. Indonesia and Germany collaboratively study tsunamis in the Indian Ocean. However, Indonesian studies focus on modeling and simulations, while German research focuses on early warning technology. This study also introduces an activity index (AI) and an attractive index (AAI) to generate time evolution trajectories of some major countries from 2000 to 2013 and evaluate their trends and performance. Four patterns of evolution are visible during this 14-year period. China and India show steadily rising contributions and impacts, USA and England show relatively decreasing research efforts and impacts, Japan and Australia show fluctuating activities and stable attraction, and Spain and Germany show fluctuating activities and increasing impacts.

## Introduction

Natural disasters have significant impacts on human society and the environment. Various researchers are studying different aspects of natural disasters. However, most existing reviews focus on partial aspects or topics of natural disaster studies. Examples are analysis methodology [1, 2], techniques [3–5], disaster risk assessment and analysis [6–8], damage assessment [9, 10], resilience [11], vulnerability [12], specific hazard [13, 14] and socioeconomic impact [15, 16]. Although Alexander [17] reviewed the natural disaster research from 1977-1997 but it did not discuss from perspectives of international collaboration or multi-disciplines. In addition, the influence of this article is lower than reviews on a subset of natural disasters research. What is the geographical distribution of natural-disaster-related research efforts? How do various countries contribute to natural disaster research and development? What subjects do the various countries and disciplines focus on? Answers to these questions are concerned by administrations, policy makers, and scientists. Hence, a comprehensive overview article of natural disaster research is required, especially from the perspectives of international and multi-disciplinary collaboration point of view.

As many reviews rely on small number of literatures read by authors and it is difficult to manually derive knowledge from the large amount of bibliometric data about whole natural disasters, complex network analysis has been used to rapidly and readily derive knowledge and discover trends and novel research topics from voluminous literature in the context of data science [18–22]. In the field of natural disaster research, for instance, the Wenchuan Earthquake research has been analyzed by using complex network analysis [23]. However, the research only focused on literature concerning an individual earthquake.

In addition, hot topics get attention from governments, corporations, and scientists for a number of years. They can indicate not only the current areas of research focus, or hotspots but also potential trends in natural disaster research. Hot topics can be identified by the number of citation or word frequencies [24]. This study used the word frequency to detect hot topics and the citation burst to show whether the hot topics get more attention within a short period.

Besides, the developing trends in different countries are illustrated by trajectories of the activity index (AI) [25] and the attractive index (AAI) [26]. The AI trajectories are used to illustrate trends in natural disaster research in a certain country. A higher AI score for a country indicates a larger relative amount of literature and more research activities in a country. The AAI trajectory is used to measure the trend of the academic impact of a country in the field of natural disaster research. A higher AAI score indicates that the country attracts more citations in this scientific field.

This study aims to gain insights into the overall natural disasters research from the perspectives of international and multi-disciplinary collaborations, by focusing on the following. The co-occurrence networks of countries and disciplines depict the cooperation and contribution of different countries and disciplines. The hot topics are detected and identified as research hotspots. The hybrid network of hot topics and countries is analyzed to find out hotspots by country. The hybrid network of hot topics and disciplines show the hotspots by discipline. Furthermore, the typical relations between these hot topics and countries are identified, as well as four developing trends of major countries in natural disaster research from 2000 to 2013 through the trajectories in the AI-AAI coordinate.

## Data and methods

Bibliographic records represent the main information of published articles. A bibliographic record contains a list of authors, the title of the article, a set of keywords, an abstract, citations etc. These records can be used to easily deduce the main ideas, study areas, research methodologies, and results of articles.

Since the focus of this study is natural disaster research in geosciences, records on natural disasters that satisfy at least one of the following three criteria were derived from the Web of Science database. Table 1 lists three classifications which were used for retrieving natural disaster literature. The first criterion was that an article was from a journal whose name contains the terms “disaster”, “disasters”, “hazard”, or “hazards”. The second criterion was that an article’s topic is related to disasters or hazards and the article was published in either Nature or Science. The third criterion was that an article’s topic is related to disasters or hazards and belonged to one of 17 categories of disaster- or hazard-related research themes. For the details of the query construction, please see S1 Appendix. of the supplementary material.

**Table 1:** List of three criteria used for retrieving natural disaster literature.

Classification	Description	Content
*PartA*	List of journals whose names contain the terms “disaster”, “disasters”, “hazard”, or “hazards”	Disasters; Natural Hazards; Disaster Advances; Natural Hazards Review; Journal of Hazardous Materials; Natural Hazards and Earth System Sciences; Disaster Prevention and Management; Geomatics Natural Hazards Risk; International Journal of Disaster Risk Science; Risk Management Journal of Risk Crisis and Disaster. Environmental Hazards-Human and Policy Dimensions; Remote Sensing: Inversion Problems and Natural Hazards; Fire and Polymers Iv Materials and Concepts for Hazard Prevention; Japca The International Journal of Air Pollution Control and Hazardous Waste Management; Journal of Environmental Science and Health Part A-ToxicHazardous Substances & Environmental Engineering; Journal Of Environmental Science and Health Part A Environmental Science and Engineering Toxic and Hazardous Substance Control.
*PartB*	Two prestigious journals	Nature; Science.
*PartC*	WOS categories	Ecology; Forestry; Geology; Limnology; Geography; Soil Science; Remote Sensing; Water Resources; Geography Physical; Environmental Studies; Engineering Geological; Environmental Sciences; Geochemistry Geophysics; Engineering Environmental; Geosciences Multidisciplinary; Agriculture Multidisciplinary; Meteorology Atmospheric Sciences.

The dataset between January 1900 and June 2015 was derived from the Web of Science(WoS) database. The titles, keywords, and abstracts of each record within the original dataset were examined by experts in order to make sure these records belong to natural disaster research. By doing so, the repeated records and articles related to hazardous materials or technical accidents (e.g. fires, explosions) were excluded manually. The final result consisted of 19694 unique records.

An article was deemed to belong to a country or a region based on the address of the corresponding author. If no corresponding author exists, the address of the first author was used. The corresponding disciplines of an article concerned were determined by its categories of WoS.

The Java application CiteSpace [27–30], which specializes in simultaneously identifying the time, frequencies, and centralities of the co-occurrence networks [31], was used to construct and visualize networks. The nodes of the networks represent countries or disciplines, which are extracted from the authors’ addresses or categories of bibliographic records. If two countries or disciplines were shown in the same bibliographic records, the link between these two nodes was constructed. Different indicators are used to visualize patterns of the generated networks. The color of the links shows the first year of the co-occurrence of the two nodes. The size of the circle is proportional to the frequency of the appearance of the literature. The frequency represents the degree of recognition in academic circles, and it reflects the academic contribution of the corresponding literature. Purple node rims indicate pivotal points with high betweenness centrality. The betweenness centrality statistics of the node act as a bridge in the development of a scientific field linking research from different time periods. The red color of the circle indicates that the node exhibits a phenomenon known as a citation burst. A citation burst occurs when the number of citations of an article or a country’s literature increases greatly within a short period.

## Results and discussion

### Natural disasters research through the eye of survey articles

Table 2 shows review articles, whose titles contain one of the following terms: disaster, disasters, hazard, hazards, natural disaster, natural disasters, natural hazard or natural hazards. Since the focus of this study is natural disaster research in geosciences, the result is refined by Web of Science categories and by manually filtering out review literatures about hazardous material and medical science. Note that the article with the highest number of citations is by Stoffel et al., published in 2008, with 158 citations.

**Table 2:** Review articles on natural disasters research and their citations (with descending order).

Authors	Title	Citations	Year
Stoffel, M.; Bollschweiler, M.	Tree-ring analysis in natural hazards research—an overview	158	2008
Alexander, D	The study of natural disasters, 1977-1997: Some reflections on a changing field of knowledge	83	1997
Meyer, V et al.	Review article: Assessing the costs of natural hazards—state of the art and knowledge gaps	82	2013
Skoufias, E	Economic Crises and Natural Disasters: Coping Strategies and Policy Implications	80	2003
Joyce, Karen E et al.	A review of the status of satellite remote sensing and image processing techniques for mapping natural hazards and disasters	71	2009
Eiser, J. Richard et al.	Risk interpretation and action: A conceptual framework for responses to natural hazards	47	2012
Bird, D. K.	The use of questionnaires for acquiring information on public perception of natural hazards and risk mitigation—a review of current knowledge and practice	47	2009
Hanewinkel, Marc et al.	Assessing natural hazards in forestry for risk management: a review	39	2011
Stoffel, M.	A review of studies dealing with tree rings and rockfall activity: The role of dendrogeomorphology in natural hazard research	33	2006
Gill, Joel C. and Malamud, Bruce D	Reviewing and visualizing the interactions of natural hazards	22	2014
Djalante, Riyanti et al	Adaptive Governance and Managing Resilience to Natural Hazards	22	2011
Dominey-Howes et al.	Queering disasters: on the need to account for LGBTI experiences in natural disaster contexts	13	2014
Garcia et al	Evaluating critical links in early warning systems for natural hazards	13	2012
Markantonis, V et al.	Valuating the intangible effects of natural hazards—review and analysis of the costing methods	11	2012
Pelling, Mark	Measuring urban vulnerability to natural disaster risk: Benchmarks for sustainability	9	2006
Xu, Lifen et al.	Natural hazard chain research in China: A review	9	2016
Papatheodorou, K et al.	An overview of the EU actions towards natural hazard prevention and management: current status and future trends	7	2014
Asgary, A. and Levy, J.	A Review of the Implications of Prospect Theory for Natural Hazards and Disaster Planning	7	2009
Gallina, Valentina et al.	A review of multi-risk methodologies for natural hazards: Consequences and challenges for a climate change impact assessment	3	2016
Glade, T et al.	An introduction to the use of historical data in natural hazard assessments	3	1999
King, Elisabeth and Mutter, John C.	Violent conflicts and natural disasters: the growing case for cross-disciplinary dialogue	2	2014
Bonaiuto, Marino at al.	Place attachment and natural hazard risk: Research review and agenda	1	2016
Klonner, Carolin et al.	Volunteered Geographic Information in Natural Hazard Analysis: A Systematic Literature Review of Current Approaches with a Focus on Preparedness and Mitigation	1	2016
Shabnam, Nourin	Natural Disasters and Economic Growth: A Review	1	2014
Finch, Kathryn C et al.	Public health implications of social media use during natural disasters, environmental disasters, and other environmental concerns	0	2016
Xu, Jiuping et al	Natural disasters and social conflict: A systematic literature review	0	2016
Komjathy, Attila et al.	Review and perspectives: Understanding natural-hazards-generated ionospheric perturbations using GPS measurements and coupled modeling	0	2016
Scoppetta, Cecilia	N̈aturald̈isasters as (neo-liberal) opportunity? Discussing post-hurricane Katrina urban regeneration in New Orleans	0	2016
Cutts, Bethany B et al.	Environmental Justice and Emerging Information Communication Technology: A Review for U.S. Natural Disaster Management	0	2015
Xi, Menghao et al.	A Review of the Methods of Natural Disaster Risk Assessment	0	2014
Yi, Hong Lei and Yang, Jay	Critical Factors for Sustainable Reconstruction Post Natural Disasters	0	2013
Hu, Hengzhi and Wen, Jiahong	A Review of Progress in Community-Based Natural Hazard Risk Analysis	0	2011
Shi, Minqi et al.	Vulnerability of Natural Disasters and An Analysis on Its conceptual Models	0	2011
Boscoianu, Mircea	Emerging Research Directions for Modeling the Impact, Short Time Recuperation and Long Term Recovery in the Case of Natural Hazards	0	2008
Poursaber, Mohammadreza et al.	Research study on appropriate interpretation techniques of satellite images for natural disaster management	0	2014

To compare with the citations of original research articles, we have listed in Table 3 the top 10 most cited research articles related to natural disaster in the resultant dataset. Their citations, including the one from the magazine *Science*, are much higher than the review articles listed in Table 2.

**Table 3:** Top 10 cited research articles related natural disaster and their citations (with descending order).

Authors	Title	Journal	Citations
Benz, UC; Hofmann, P; Willhauck, G; Lingenfelder, I; Heynen, M	Adaptation, adaptive capacity and vulnerability	Global environmental change-human and policy dimensions	827
Guzzetti, F; Carrara, A; Cardinali, M; Reichenbach, P	Multi-resolution, object-oriented fuzzy analysis of remote sensing data for GIS-ready information	ISPRS Journal of photogrammetry and remote sensing	770
Adger, WN; Hughes, TP; Folke, C; Carpenter, SR; Rockstrom, J	Progressive failure on the North Anatolian fault since 1939 by earthquake stress triggering	Geophysical journal international	520
Boore, DM	Social-ecological resilience to coastal disasters	Science	386
Carrara, A; Cardinali, M; Detti, R; Guzzetti, F; Pasqui, V; Reichenbach, P	Simulation of ground motion using the stochastic method	Pure and applied geophysics	357
Ayalew, L; Yamagishi, H	GIS techniques and statistical-models in evaluating landslide hazard	Earth surface processes and landforms	344
Hastenrath, S; Heller, L	The application of GIS-based logistic regression for landslide susceptibility mapping in the Kakuda-Yahiko Mountains, Central Japan	Geomorphology	313
Benz, UC; Hofmann, P; Willhauck, G; Lingenfelder, I; Heynen, M	Dynamics of climatic hazards in northeast Brazil	Quarterly journal of the royal meteorological society	312
De Vivo, B; Rolandi, G; Gans, PB; Calvert, A; Bohrson, WA; Spera, FJ; Belkin, HE	New constraints on the pyroclastic eruptive history of the Campanian volcanic Plain (Italy)	Mineralogy and petrology	305
Dai, FC; Lee, CF	Landslide characteristics and, slope instability modeling using GIS, Lantau Island, Hong Kong	Geomorphology	281

In general, most of the review articles focused on some specific aspects or topics of natural disasters research, such as analysis methodology, techniques, disaster risk assessment and analysis, damage assessment, resilience, vulnerability, specific hazard and socioeconomic impact. In particular, few review articles paid attention to the subject related to the international or multidisciplinary cooperations. This results in their low number of citations and low impact in natural disasters research. We hope that the co-occurrence network taking countries or disciplines as nodes can be efficient and objective for illustrating the cooperational relationships among different countries or different disciplines.

### Spatial distribution of natural disaster research

**Fig 1:**
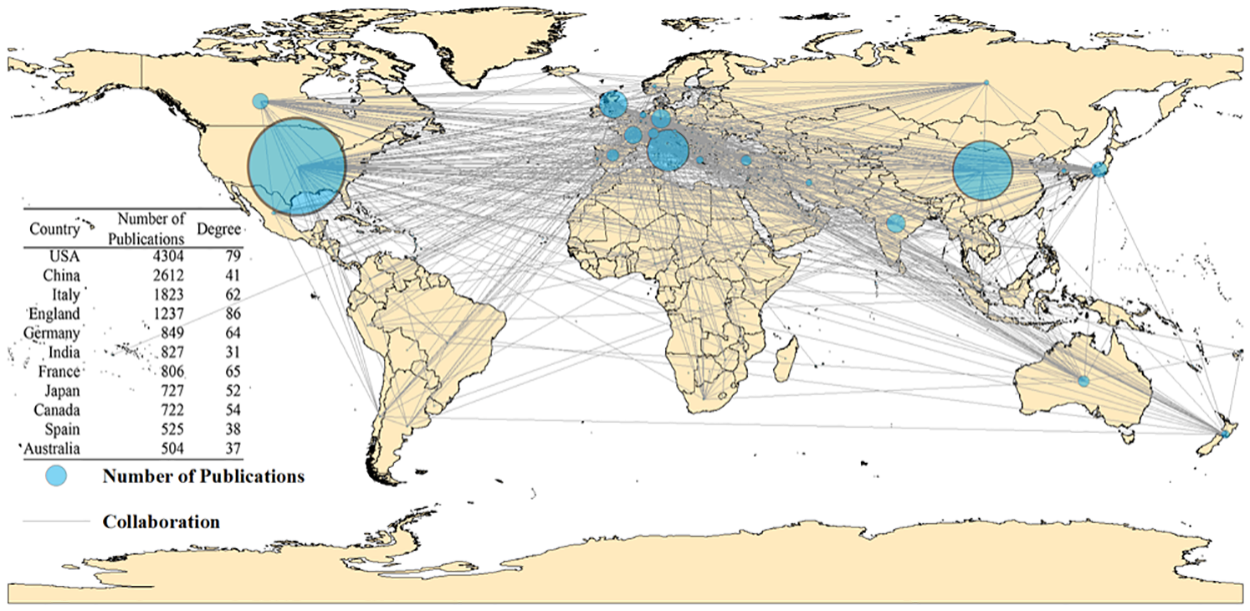
Geographic distribution of collaborating countries. Circular nodes represent countries/regions. The size of a circle is in proportion to the number of literatures of the country. The links of nodes represent cooperating relations between countries or regions.

The network of collaborating countries was constructed and visualized using CiteSpace. To derive high impact articles from the dataset, a modified g-index [32] was employed to filter out insignificant publications. The dataset was sliced into 1-year slices from 1900 to 2015. Published articles and their information were selected if their modified g-indexes were 5 or higher. These articles were then represented geographically based on the countries’ coordinates using Gephi [33], as shown in Fig 1.

Fig 1 presents the global spatial distribution of collaboration on natural disaster research during the period from 1900 to 2015. The node size indicates the number of natural disaster publications of that country. The “degree” column in Fig 1 represents the number of links between the reference country and all other countries. For example, the number 67 for USA means its researchers collaborate with colleagues in 67 other countries. The geographic distribution of collaborating countries falls into four groups, essentially aligning a country to the continent it belongs, i.e., North America, Europe, Asia, and Oceania.

North America has three significant members, which are USA, Canada, and Mexico. According to the number of publications, USA is not only the continent’s leading nation in natural disaster research but also the dominant country worldwide. The dense links with Europe show that USA cooperates intensively with European countries. China, India, and Japan are the three major countries in the Asia group and China is more active than other countries in the region. The degrees of China and India are lower than that of Japan in terms of their numbers of publications. The Oceania group, which includes Australia and New Zealand, has patterns similar to that of Japan, which are a small amount of articles and relatively great degrees.

**Fig 2:**
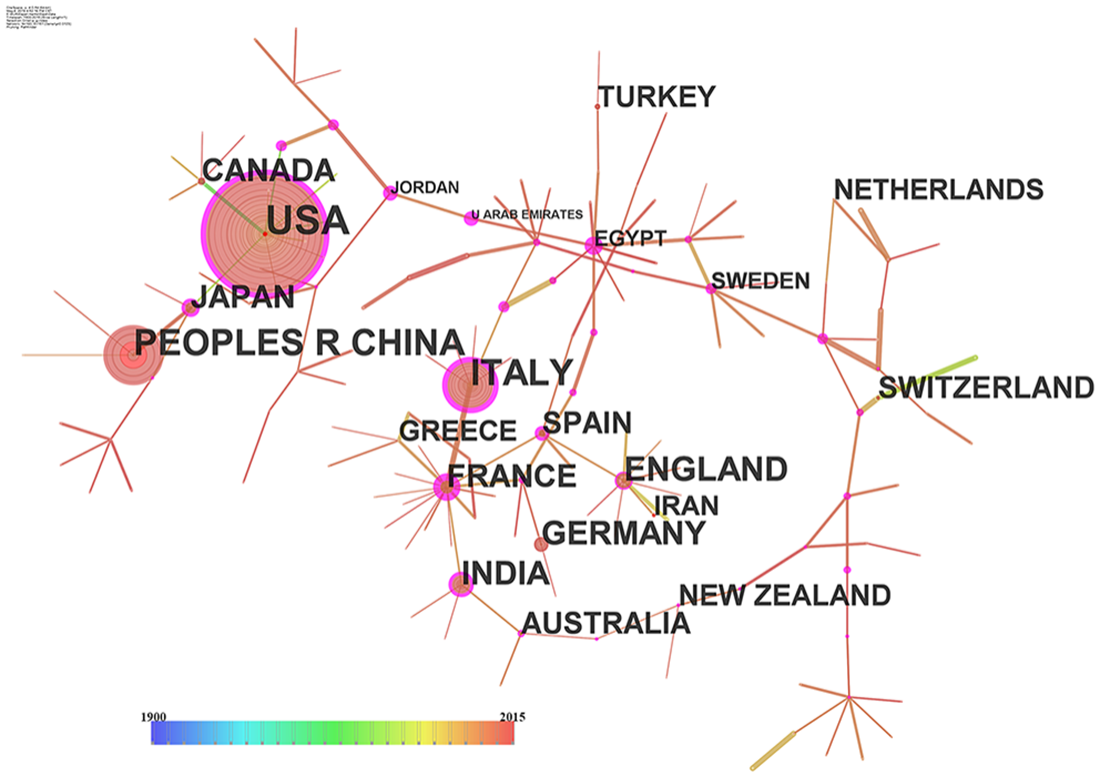
A collaboration network of natural disasters of 160 nodes and 157 links. Circular nodes represent countries/regions. The size of a circle is in proportion to the number of literatures of the country. The colors of rings of a circle are corresponding to the year. The purple rims of circles represent the high betweenness centralities.

In order to investigate the details of collaborations, the pathfinder algorithm was used to prune each merged network to improve the clarity of the resultant network. Fig 2 shows the pruned collaborating network consisting of 160 nodes and 157 links.

In the networks, nodes with a purple ring depict potentially pivotal nodes with high betweenness centralities [34]. Among the countries with high betweenness centralities in the collaboration network, USA, China, and Italy are prominent countries with significant volume. USA published 4304 articles, compared to the 2612 articles published by China, and the 1823 articles published by Italy. The developed countries with significant contributions to natural disaster studies include England (1237), Germany (849), France (806), Japan (727), Canada (722), Spain (525), Australia (504), Switzerland (453), Turkey (439), New Zealand (453), Greece (344), and the Netherlands (309). Europe is the most significant contributor to natural disaster research. More than half the active countries come from this region. Further, European countries collaborate with each other intensively. Except China, India and Iran are two other major developing countries that have made significant contributions to natural disaster research, with 827 and 282 published articles, respectively.

### The hot topics of natural disaster research

Burst detection [35] was employed to detect hot topics from noun phrases of titles and keywords in terms of their frequency. 144 hot topics were detected and classified into four categories. The first category is associated with specific natural disasters or hazards (e.g. Haiti earthquake, Indian Ocean tsunami, Wenchuan earthquake, and lava flows). The second category is conceptual, e.g., social vulnerability. The third is associated with methods, e.g., prediction models and the support vector machine. The fourth category is the class of a study area or object, e.g., New Zealand.

Furthermore, Table 4 lists the top ten frequent hot topics. The strength in Table 4 indicates if the hot topics have citation burst. Four hot topics are detected because of exhibiting citation bursts. Strength indicates the rate of growth of the hot topics’ citations. The year represents the first appearance of the hot topic. For instance, “Indian Ocean tsunami” first appears in 2004, indicating that a great tsunami occurred in that year. It experienced rapid growth from 2005 to 2006. In recent years (2013-2015), “social vulnerability”, “prediction model” as well as “landslide inventory map” have experienced citation bursts, which means they have drawn significant attention from scientists.

**Table 4:** The list of three classifications used for retrieving natural disaster literature.

Frequency	Strength	Label	Year
61	/	high-resolution	2009
28	/	wenchuan-earthquake	2009
21	12.82	social-vulnerability	2013
17	/	active-faults	2001
17	/	practical-implications	2009
17	10.27	prediction-model	2013
16	9.64	landslide-inventory-map	2013
15	/	peak-ground-acceleration	1999
13	/	fault-zone	2000
13	8.76	indian-ocean-tsunami	2006

‘/’ indicates no burst phenomenon.

**Fig 3:**
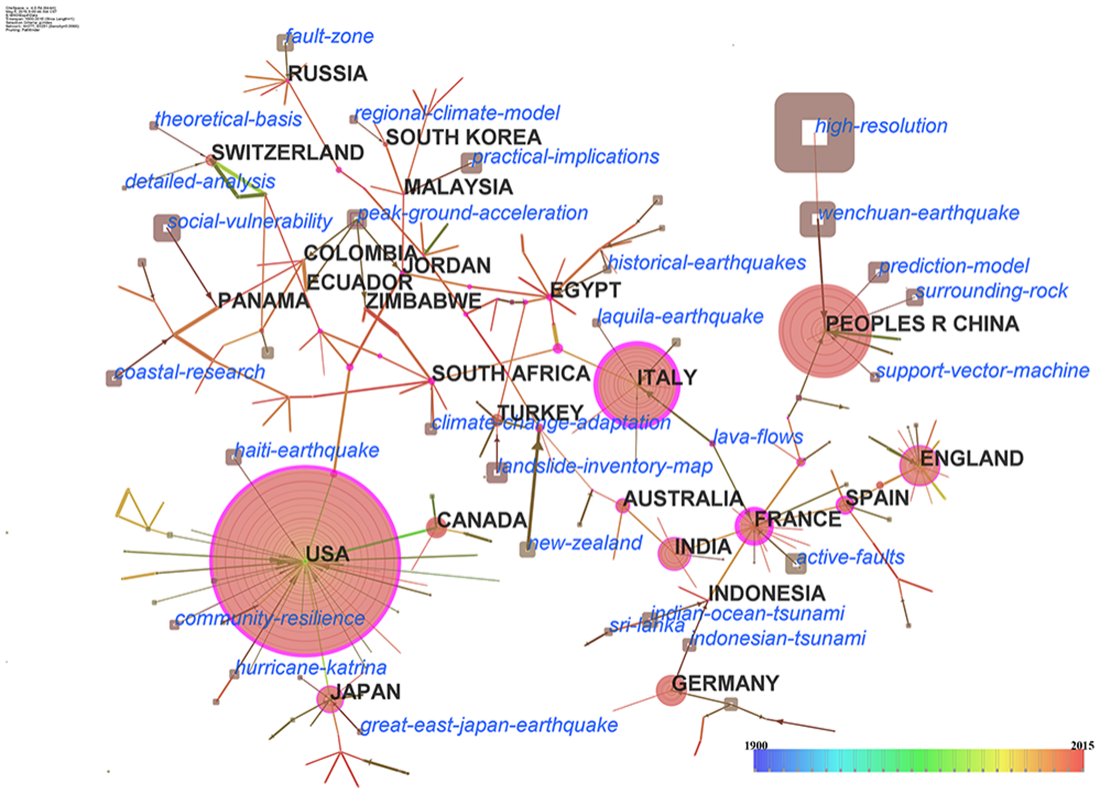
A hybrid network of countries (regions) and hot topics of 277 nodes and 251 links. Circular nodes represent countries/regions. Square nodes represent topics. The color of the ring of a circle are corresponding to the year. The size of a circle is in proportion to the number of literatures of the country. The size of a square is in proportion to the frequency of the topic. The purple rims of nodes represent the high betweenness centralities.

A hybrid network, whose nodes represent countries and hot topics, was generated by choosing countries and terms as the nodes of co-occurrence network in CiteSpace (Fig 3). In Fig 3 each blue label represents an associated hot topic and is attached to a country or region. The size of the node is proportional to the frequency of appearance of the terms.

Links between major natural disasters and countries were investigated. For example, the Wenchuan earthquake points to the node labeled “PEOPLES R CHINA”, indicating that researchers from China paid a lot of attention to this disaster. Similar links are investigated, such as those between ‘Haiti earthquake’ and USA, ‘Great East Japan earthquake’ and Japan, ‘Laquila earthquake’ and Italy, and ‘Indian Ocean tsunami’ and Indonesia.

These phenomena can be explained by the spatial connections of the disaster events and the relevant countries. The Wenchuan earthquake occurred in Sichuan province in China. The Haiti earthquake occurred in Haiti on 2010. Although Haiti is located on the island of Hispaniola, the Haiti earthquake drew a lot of attention from USA because of its geographical proximity to Haiti. The Great East Japan earthquake was an undersea earthquake that occurred off the coast of Japan in 2011. L’Aquila is located in central Italy. The most recent major earthquake that occurred in L’Aquila was the one in 2009. The Indian Ocean tsunami was a severe tsunami caused by a strong earthquake that occurred off the west coast of Indonesia.

Additionally, there are links between concepts and countries, such as South Korea and ‘region climate model’, Panama and ‘social vulnerability’, South Africa and ‘climate change adaptation’, and Malaysia and ‘practical implications’. These links indicate the research hotspots of these countries. South Korean scientists focus on regional climate models. Social vulnerability is a hotspot in Panama. South African researchers are concerned with research on climate change adaptation. Malaysian scientists focus on practical implications.

**Fig 4:**
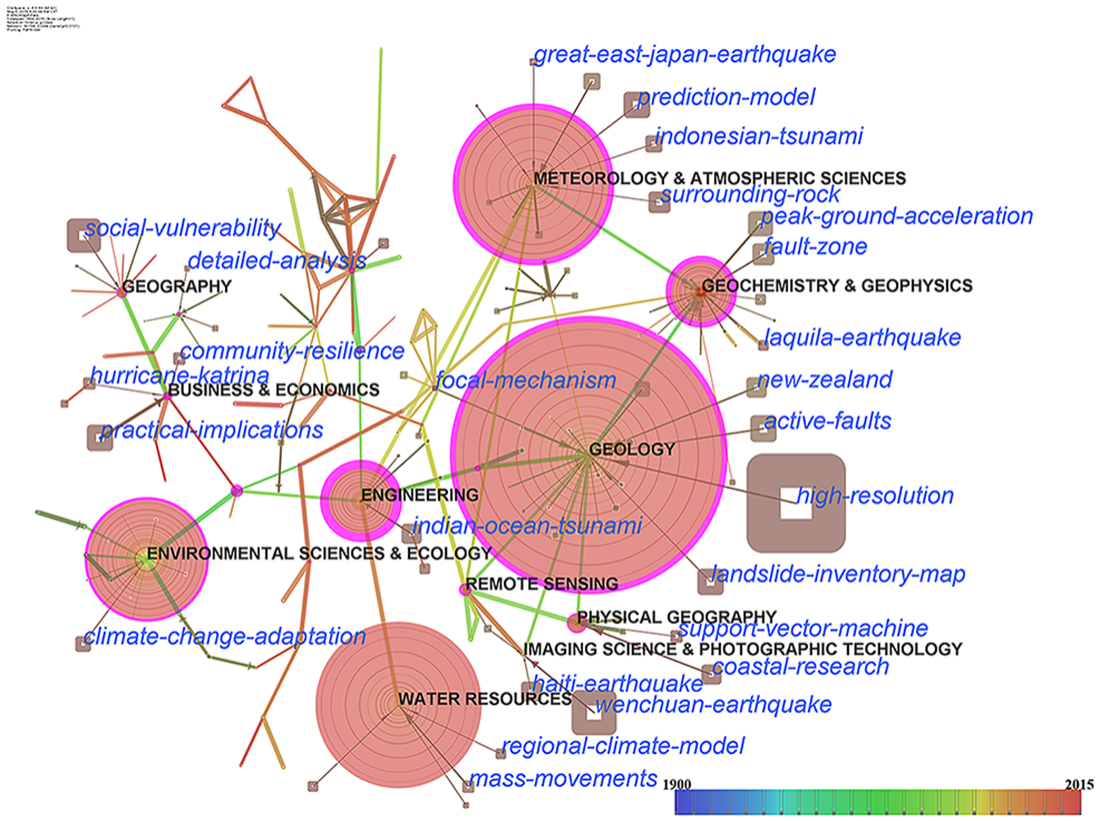
A hybrid network of disciplines and hot topics of 194 nodes and 246 links. Circular nodes represent disciplines. Square nodes represent topics. The color of the ring of a circle is corresponding to the year. The size of a circle is in proportion to the number of literatures of the discipline. The purple rims of circles represent the high betweenness centralities. The size of a square is in proportion to the frequency of the topic.

By using the same method of Fig 3, the hybrid network of disciplines and hot topics for the period from 1900 to 2015 was built (Fig 4). The time span was set as 1 year. The g-index filter was 5. The resultant hybrid network was also pruned by pathfinder algorithm.

Fig 4 illustrates connections between hot topics and disciplines. From the disciplines perspective, geology exhibits the highest frequency of publications appearances and high betweenness centrality. The high betweenness and size of the geology node indicate that geology plays a core and pivotal role in natural disaster research. Environmental sciences, ecology, geochemistry, geophysics, engineering, water resources, meteorology, and atmospheric sciences have significant contributions to natural disaster research.

In addition to the hybrid network of hot topics and countries, various hotspots of different disciplines are identified. In Fig 4, both the Wenchuan and Haiti earthquakes are attached to the disciplines of imaging science and photographic technology [36–38]. The Laquila earthquake points to geochemistry and geophysics [39, 40]. To study earthquakes, researchers from imaging sciences focus on applying imagery data and methods, and researchers from geochemistry or geophysics prefer to use methodologies related to their own expertise. The Indian Ocean tsunami links to the node labeled engineering [41, 42]. However, the Indonesian tsunami connects to meteorology and atmospheric sciences. This indicates that different disciplines are interested in the engineering and meteorology aspects of the tsunami. Environmental scientists and ecologists pay attention to climate change adaptation. Social vulnerability is a hotspot in geology and related disciplines. Geologists are more concerned about the topic of landslide inventory mapping. Meteorology and atmospheric sciences pay a lot of attention to research on prediction models.

A comparison between two hybrid networks of hot topics and countries provides insight into the differences in research focus. The Wenchuan and Haiti earthquakes connect to China and USA respectively. However, these two topics link to the same disciplines. This indicates that researchers from the both countries apply imaging science and photographic technology to study earthquakes [36–38]. The links of the Laquila earthquake to Italy and disciplines indicate that the research associated with the Laquila earthquake is conducted using geochemical or geophysical methods in Italy [39, 40]. The terms ‘Indian Ocean tsunami’ and ‘Indonesian tsunami’ representing the same catastrophe are related to engineering and the disciplines of meteorology and atmospheric sciences respectively. The relations indicate that, in the immediate aftermath of the disaster, Indonesian scientists concentrated on the engineering impacts and reconstruction of the tsunami [41, 42]. Later, researchers from Indonesia and Germany collaboratively studied the Indian tsunami but focused on model simulation [43] and early warning technology [44, 45], respectively.

## The development of selected countries in natural disaster research

To evaluate the efficiency and temporal changes of countries, we employed two indexes, i.e., the activity index (AI) and the attractive index (AAI). The activity index is an indicator of the relative effort devoted by a country to a research field, while the attractive index indicates the relative impact made by a country in terms of attracting citations through its publications [26]. The AI and AAI are applied and transformed as follows [46]:

$$\begin{array}{c} AI_{i}^{t}=\frac{P_{i}^{t}/\sum P}{TP^{t}/\sum TP} \end{array}$$ (1)

$$\begin{array}{c} AAI_{i}^{t}=\frac{C_{i}^{t}/\sum C}{TC^{t}/\sum TC} \end{array}$$ (2)

where $AI_{i}^{t}$ is the activity index of country *i* in the year *t*; $P_{i}^{t}$ is the number of articles on natural disasters published by country *i* in the year *t*; ∑*P* is the total natural disaster publications of country *i* during the period of publication; *TP*^*t*^ is the global natural disaster publications in the year *t*; ∑*TP* is the sum of the global natural disaster publications during a period. Similarly, $AAI_{i}^{t}$ indicates the attractive index of country *i* in the year *t*; $C_{i}^{t}$ is the citations of natural disaster publications of country *i* in the year *t*; ∑*C* is the sum of citations of natural disaster publications of country *i* during a period; *TC*^*t*^ represents the global natural disaster citations in the year *t* and ∑*TC* is the total natural disaster citations during the same period as that of ∑*C*.

*AI* = 1 and *AAI* = 1 represent the global average level of natural disaster research effort and academic impact, respectively. *AI* > 1 or *AI* < 1 indicates that a countrys research effort higher or lower than the global average; *AAI* > 1 or *AAI* < 1 indicates that the number of citations attracted by a country is more or less than the global average level of citations.

**Fig 5:**
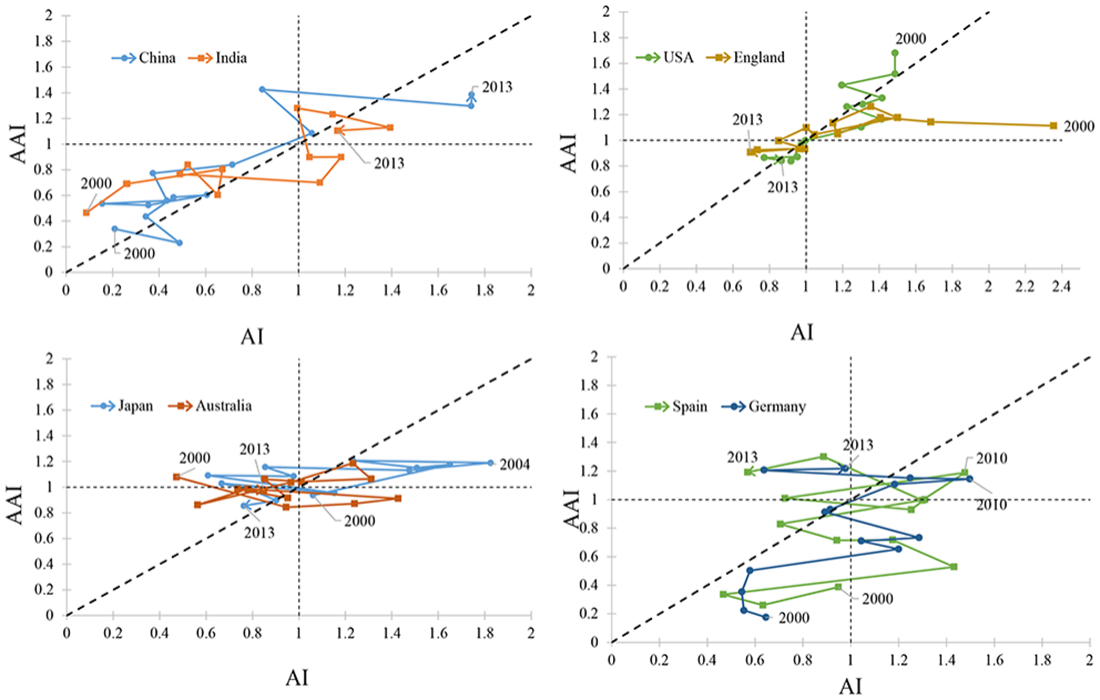
Relational chart of AI and AAI from 2000 to 2013 for 8 countries/regions, China, India, the U.S., England, Japan, Australia, Spain, and Germany. The reference line *x* = *y* represents the balanced status of AI and AAI of a country/reigon’s natural diaster research.

It should be noted that it is very difficult that publications attract citations in the first year of publishing. There is generally a lag between the time of publication of an article and the time of citations [47, 48]. Considering this fact, the time scope of the activity index and attractive index are set as 2000 to 2013 and 2002 to 2015, respectively. The activity index and attractive index of eight selected countries were calculated(see S1 Table.) and presented in the form of a relational chart (Fig 5). The reference line (*y* = *x*) reflects a situation in which a country’s research effort is balanced with their impact of citations in natural disaster research.

These selected countries experienced four types of evolutions. China and India increased significantly since 2000. Their efforts on natural disaster research grew continuously. In contrast, USA and England showed a relative decrease to a level slightly lower than the global average. Simultaneously, they approach the reference line, which means that their research efforts are balanced with the impact of citations. The efforts from the other four countries, Japan, Australia, Spain, and Germany, fluctuate from 2000 to 2013. The difference is that Japan and Australia maintain a relatively high attraction and the impacts of Spain and Germany increase.

## Conclusion

Natural disaster-related literature from 1900 to 2015 was derived from the Web of Science database. Using CiteSpace and Gephi, complex networks were generated to visualize and analyze spatial patterns, collaborations, and research hotspots of countries and disciplines in natural disaster research.

The geographic distribution analysis confirms that the research on natural disasters is a global scientific field. USA, China, and Italy are the three most productive countries in terms of natural disaster research. Developed countries account for most of the outputs and exhibit more international cooperation than developing countries.

The analysis of hot topics shows that the frequency and citation of emerging disaster events grow rapidly after it occurs, which reflects the great influence of catastrophes on academia. The four hot topics with strong citation bursts are detected to represent not only research hot topics of the concerned countries and disciplines but also the potential trend of natural disaster research. The three hot topics “social vulnerability”, “prediction model”, and “landslide inventory map” with strong citation bursts from 2013 to 2015 indicate the trend of natural disaster research as well.

The relations among hot topics, countries, and disciplines provide research hotspots of countries and disciplines in natural disaster research. From the country perspective, natural disaster research is a regional research field. The connections between catastrophes and countries indicate that great natural disasters or catastrophes are gained more attention from the countries in which they occur or neighboring countries. Although the countries with large numbers of publications play important roles, small countries, such as Panama and Turkey, are still investigated to focus on hot topics in this field. From the discipline perspective, the hotspots of different disciplines were investigated. The results show that natural disaster research is a typical multidisciplinary research field. The same type of natural disasters or catastrophes can attract attention from various disciplines.

Furthermore, more details and integrated information of natural disaster research are shown by combining the two hybrid networks. For example, both USA and China employ imaging science to the earthquake research field. Indonesia focused on the engineering aspects of the tsunami-hit and recently collaborated with Germany on modeling and simulation. However, Germany prefers the technology of the tsunami early warning system.

In addition, using the AIs and AAIs, the comparison of countries performance indicates that China and India have achieved great progress and become the new significant contributors in natural disaster research since 2000. Their influences are greater the worlds average since 2010. This means that the role of developing countries is more important than in the past The research effort and impacts of developed countries relatively reduced (USA and England), or fluctuated (Japan, Australia, Spain, and Germany) in the same period. However, developed countries still have significant impacts in this field.

In this study, we focused on the developing trend and hot topics of natural disaster research and its relationship with neighborhood social conditions, whereas data extraction methods, the data accuracy, and the topic identification method were not fully considered in this study. Future studies may include these factors to explore more accurate data extraction method, compare the influence of different method of topic identification and data retrieval.

## Supporting information

S1 Table (XLSX)

S1 Appendix (DOC)
