# Supplementary material for: Visualized analysis of developing trends and hot topics in natural disaster research
Source: PLoS One. 2018 Jan 19;13(1):e0191250. doi: 10.1371/journal.pone.0191250 (PMC5774750; doi:10.1371/journal.pone.0191250)
Supplement: S1 Appendix — (DOC) [file pone.0191250.s002.doc]

**Appendix. Web of Science Query Construction**

The first query used to retrieve natural disaster research from journals is shown as follows:

SO=("Natural Hazards" OR "Disasters" OR "Disaster Advances" OR "International Journal of Disaster Risk Science" OR "Environmental Hazards-Human and Policy Dimensions" OR "Natural Hazards and Earth System Sciences" OR "Natural Hazards Review " OR "Disaster Prevention and Management" OR "Geomatics Natural Hazards Risk" OR "Geomatics Natural Hazards & Risk" OR "Journal Of Hazardous Materials" OR "Journal Of Environmental Science And Health Part A Environmental Science And Engineering Toxic And Hazardous Substance Control" OR "Japca The International Journal of Air Pollution Control And Hazardous Waste Management" Or "Risk Management Journal Of Risk Crisis And Disaster")

The second query is used for retrieving natural disaster research from Nature and Science;

SO= (“Nature” OR “Science”) AND TS= (disaster$ OR hazard$)

The last query is used for searching natural disaster research from disaster-related categories of WoS.

TS= (disaster$ OR hazard$) AND WC= ("ECOLOGY" OR "ENVIRONMENTAL SCIENCES" OR "METEOROLOGY ATMOSPHERIC SCIENCES" OR "WATER RESOURCES" OR "FORESTRY" OR "GEOSCIENCES MULTIDISCIPLINARY" OR "AGRICULTURE MULTIDISCIPLINARY" OR "GEOCHEMISTRY GEOPHYSICS" or "SOIL SCIENCE" or "GEOGRAPHY PHYSICA" OR "ENVIRONMENTAL STUDIES" OR "ENGINEERING GEOLOGICAL" or "LIMNOLOGY" OR "ENGINEERING ENVIRONMENTAL" OR "GEOLOGY" OR "REMOTE SENSING" OR "GEOGRAPHY")
